# Multiple Papillomatosis of Breast and Patient's Choice of Treatment

**DOI:** 10.4061/2010/540590

**Published:** 2010-12-26

**Authors:** Debasish Debnath, Dhafir Al-Okati, Wael Ismail

**Affiliations:** ^1^Department of Surgery, Queen's Hospital, Barking, Havering, and Redbridge University Hospitals NHS Trust, Essex RM7 0AG, UK; ^2^Department of Pathology, Queen's Hospital, Barking, Havering, and Redbridge University Hospitals NHS Trust, Essex RM7 0AG, UK

## Abstract

Papillary lesions of breast represent a range of lesions. Intraductal papilloma and its association with nipple discharge are well known. However, multiple papillomatosis has quite distinct characteristics and decision making can be somewhat challenging. We report a case of multiple papillomatosis in association with ductal carcinoma in situ (DCIS). Patient opted for ipsilateral mastectomy and prophylactic mastectomy of contralateral breast. Her decision of having prophylactic mastectomy was vindicated by presence of incidental DCIS in the contralateral breast. To our knowledge, this is the first reported case of multiple papillomatosis with DCIS of breast, along with incidental synchronous papillomatosis of contralateral breast with DCIS. The case illustrates few distinct features of multiple papillomatosis of breast and exemplifies how a patient's choice is so paramount in decision making process. Patients should be fully informed of the treatment options of the condition, and their wishes should be fully taken into account while making the final decision.

## 1. Introduction

Papillary lesions of breast have varied morphological, radiological, and pathological features. Such lesions are characterized by formation of epithelial fronds that have both the luminal epithelial and the outer myoepithelial cell layers, supported by a fibrovascular stroma [1]. Papillomas of the breast can be divided into solitary papillomas, juvenile papillomatosis, and multiple papillomatosis [2]. Their malignant potentials vary and may have an impact on patients' decision making process. We report a case of multiple papillomatosis of breast where patient's choice of treatment was affected by the anxiety of risk of malignancy and decided to opt for prophylactic mastectomy. 

## 2. Case Report

A 41-year-old woman presented with chief complaint of a lump in right breast of eight weeks duration. Her maternal aunt had breast cancer at age fifty-five. On examination, a firm 2.5 × 2 cm^2^ mass was felt in upper outer quadrant (UOQ) of right breast.

**Figure 1:**
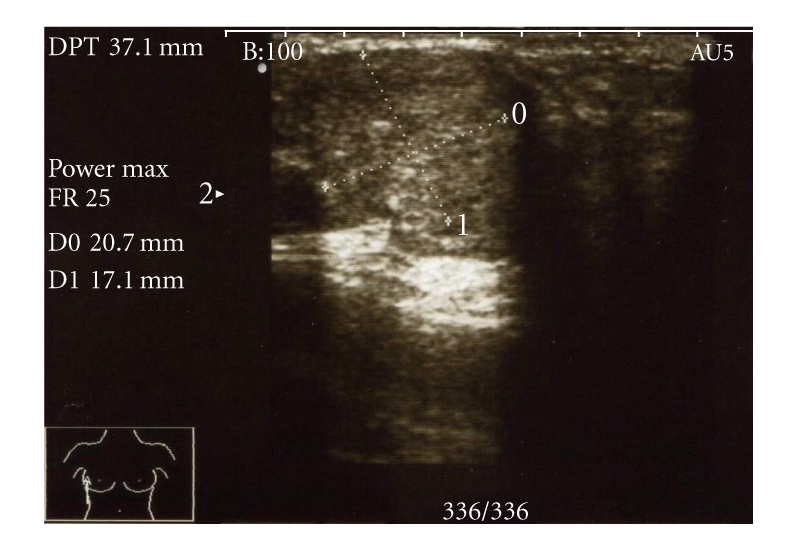
Ultrasound scan of the upper outer quadrant lesion of the right breast.

Mammogram showed round soft tissue opacity in OUQ of right breast (M2). Ultrasound scan showed a solid nodule suggestive of fibroadenoma measuring 21 × 17 × 17 mm^3^ in the lateral aspect (U3) of right breast (Figure 1).

Fine-needle aspiration cytology of UOQ lesion of the right breast was noted to be C4, but core biopsy showed benign papillary proliferation (B3). Excision biopsy of the lesion demonstrated multiple papillomatosis containing areas of atypical ductal hyperplasia and DCIS, forming a cribriform pattern (20 mm in size). The excision was stated to be incomplete.

The results were discussed with patient. She was explained about the risk of developing cancer in future, and various options were given. She requested for bilateral mastectomy (prophylactic on left side). Following appropriate psychological assessment, she underwent bilateral mastectomy along with immediate breast reconstruction using expander prosthesis. Her postoperative recovery was uneventful.

**Figure 2:**
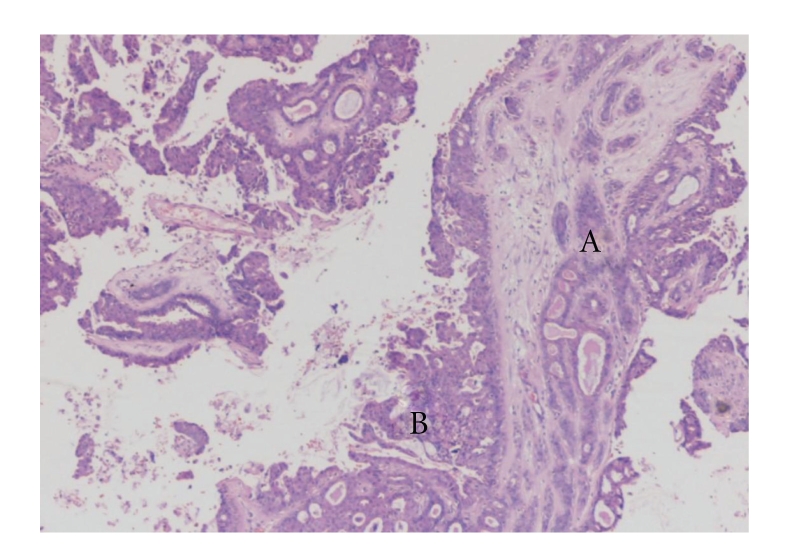
Microphotograph of histology of the symptomatic upper outer quadrant lesion (Haematoxylin & Eosin ×4) of the right breast (A = multiple papillomatosis, B = low grade DCIS).

**Figure 3:**
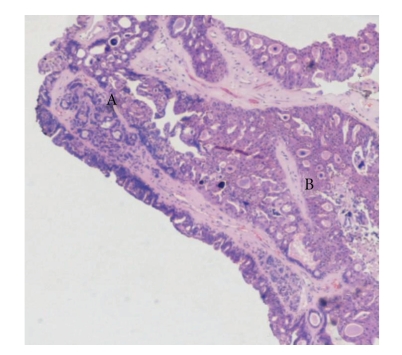
Microphotograph of histology of the incidental upper outer quadrant lesion (Haematoxylin & Eosin ×4) of the left breast (A = papillomatous lesion, B = low grade DCIS).

Histology of right mastectomy specimen showed residual multiple papillomatosis with low-grade DCIS (Figure 2). Taking previous excision into consideration, the sizes of papillary lesion and DCIS amounted to 24 mm and 32 mm, respectively. Histology of left mastectomy specimen showed an area of papillomatous lesion (9 mm) in the upper outer quadrant of breast with transition to low-grade DCIS (15 mm) (Figure 3). Excision was complete on both sides. No invasive malignancy was noted on either side.

## 3. Discussion

Pathologically, a papilloma is a mass-like projection that consists of papillary fronds attached to the inner mammary duct wall by a fibrovascular core that is covered with ductal epithelial and myoepithelial cells [2]. The epithelial component can be subject to a spectrum of morphologic changes ranging from metaplasia to hyperplasia, atypical intraductal hyperplasia, and in situ carcinoma [2, 3].

Intraductal papilloma is a discrete benign tumour of the epithelium of mammary ducts. It shows a predilection for the extreme ends of the ductal system the lactiferous sinuses and the terminal ductules [4]. Clinically, solitary papillomas commonly occur in perimenopausal women, who usually present with spontaneous nipple discharge. Solitary papillomas are associated with a slightly increased risk (1.5–2.0 times) of developing breast carcinoma [5]. A solitary papilloma occasionally appears on mammography as a circumscribed subareolar mass or as a solitary dilated retroareolar duct [2]. On sonography, a papilloma is seen as an intraductal mass in a dilated duct, an intracystic mass, or a solid mass with a well-defined border [5]. Ductography may show an intraluminal filling defect or ductal dilatation due to partial or complete ductal obstruction. Recently, MRI has been reported to be an additional useful technique for detecting intraductal papillomas of breast [6].

On the other hand, papillomatosis is defined as a minimum of five clearly separate papillomas within a localized segment of breast tissue [1]. Juvenile papillomatosis (JP) of the breast is defined as severe ductal papillomatosis occurring in women less than thirty years old. Pathologic findings consist of papillomatosis and extensive cyst formation [7]. Patients typically present with a painless, circumscribed, mobile mass, which is easily confused with a fibroadenoma [7]. As the lesions usually occur in young women, patients are usually first evaluated with sonography. Juvenile papillomatosis is seen on sonography as an ill-defined heterogeneous mass with multiple peripheral small cystic spaces. Mammography usually shows dense breast tissue with no detectable lesion or asymmetric density [8]. Follow-up studies have suggested that JP is associated with an increased risk of breast cancer. Patient's female relatives and the patient herself may be at increased risk for developing carcinoma, particularly if the lesion is bilateral and the patient has a family history of breast cancer [9–11]. Therefore, long-term followup is recommended both for the patient and the family [10, 12].

Multiple papillomatosis occurs in approximately 10% of cases of intraductal papillomas, tends to occur in younger patients than solitary papillomas, and is usually peripheral in location. Multiple papillomatosis arises in the terminal ductal lobular units and are more frequently associated with hyperplasia, atypia, DCIS, sclerosing adenosis, and radial scar [3]. Cardenosa et al. noted that the incidence of atypical ductal hyperplasia was 43 percent [13]. Clinically, patients commonly present with palpable masses [2]. Nipple discharge is less commonly seen, occurring as the presenting complaint only in approximately 20% of patients [13]. The patient in this case presented with a lump in the breast, and there was no history of nipple discharge. Mammographic findings of multiple papillomatosis are variable and include round, oval, or slightly lobulated well-circumscribed or spiculated masses with or without calcification, foci of microcalcification, clusters of nodule, and asymmetric density. On sonography, multiple papillomatosis lesions are seen as round, oval, or lobulated circumscribed solid masses or complex masses [14]. Indeed, mammogram in the case presented showed round soft tissue opacity and the ultrasound showed a solid lesion. However, early or small lesions may remain radiologically occult. Micropapillary DCIS without calcification is difficult to recognize on mammography, or findings may be nonspecific. This would explain the lack of radiological signs of “incidental” lesions noted in the left breast in the case reported [2, 13].

Bilateral disease and recurrences after surgical treatment are more common in multiple papillomatosis [14, 15]. Association of multiple papillomatosis with in-situ carcinomas ranged from 10% to 37.5% [15–17]. Actual size of the lesion may be larger than that appreciated by clinical and imaging findings. This may give rise to the potential of incomplete excision and recurrence. In one series, bilateral disease was reported in as many as 14% of patients, and 24% had recurrences after surgical treatment [15]. Invasive carcinoma has also been noted, but is rare. Therefore, if treated conservatively (i.e., wide local excision), patients with multiple papillomatosis should be kept under annual review. In addition to digital mammography, magnetic resonance can be also used in surveillance in view of its high sensitivity in detecting papillomas and demonstrating multicentric nature of the disease [18, 19].

Prognosis of papillary lesions, even though associated with DCIS, remains excellent. Even those with papillary carcinoma have a better prognosis, with less axillary nodal involvement, than those with other forms of ductal carcinomas [3, 20, 21]. Multifocality and sizes of lesions, associated risk factors and patients' wishes may help decide the type of surgery, such as breast conservative surgery or mastectomy. It should be emphasised that patients who undergo breast conservative surgery do not necessarily have a worse prognosis than patients treated by a mastectomy. It is recommended that if breast conservative treatment is undertaken, a clear margin of at least 10 mm should be adhered to [22, 23].

Patient's choice in decision making is paramount in such cases, as demonstrated in the report. As part of the informed consent process, patients must receive sufficient information, in a way that they can understand, to enable them to exercise their right to make informed decisions about their care [24].

The patient under consideration could not cope with the thought of the risk of papillomatosis (and hence, cancer) to the contralateral breast and opted for bilateral mastectomy. Her decision was vindicated by the finding of incidental DCIS on the asymptomatic side (left breast). We have not come across any previous reported case of multiple papillomatosis with DCIS of breast, along with incidental synchronous papillomatosis of contralateral breast with DCIS, which makes this case unique.

## 4. Conclusions

Clinicians should be aware of various papillary lesions of breast. Multiple papillomatosis of breast remains a distinct entity, has a high propensity of being bilateral and recurrent, and is associated with in-situ carcinomas. It is important to emphasise such characteristics to the patient while discussing treatment options so that patient can make an informed choice.
